# Adaptability and responsiveness: keys to operational measures in a regional hospital radiology department during the current COVID-19 pandemic

**DOI:** 10.1259/bjro.20200017

**Published:** 2020-06-19

**Authors:** Pratik Mukherjee, Tze Chwan Lim, Ashish Chawla, Hong Chou, Wilfred C G Peh

**Affiliations:** 1 Department of Diagnostic Radiology, Woodlands Health Campus, Singapore; 2 Department of Diagnostic Radiology, Khoo Teck Puat Hospital, Singapore

## Abstract

The rapid and mostly uncontrolled spread of the coronavirus disease 2019 pandemic over the past 4 months has overwhelmed many healthcare systems worldwide. In Singapore, while our public healthcare institutions were considered well prepared due to our prior experience with the SARS outbreak, there was an unexpected surge of infected patients over the recent 2 months to deal with. We describe our radiology department’s experience in modifying operational practices and implementing strict infection control measures aimed at minimizing disease transmission and mitigating the potential impact of possible staff infection. From the perspective of serving a medium-sized regional hospital and limited by physical and manpower constraints, our radiology department had to adapt quickly and modify our initial responses and practices as the disease scenario changed. We have also enumerated some guidelines for planning future radiology departments.

## Introduction

The end of December 2019 saw the emergence of a previously unknown viral pneumonia in Wuhan, capital of Hubei province in China. On 2 January 2020, the Singapore Ministry of Health (MOH) issued a public notification of this infection cluster in Wuhan. The World Health Organization (WHO) declared the novel coronavirus disease 2019 (COVID-19) outbreak as a Public Health Emergency of International Concern on 30 January 2020 and subsequently, classified it as a pandemic on 11 March 2020. As of 31 May 2020, there were 6 million infected people around the globe across 212 countries and territories, with 371,471 dead.^1^

The 2003 severe acute respiratory syndrome (SARS) outbreak in Singapore, while resulting in only 238 infected patients over a 3-month period, caused 33 deaths, with a transmission rate of 40.8% among healthcare workers (HCWs).^2^ Building upon this past experience, pandemic preparedness in the Singapore, particularly in the public health institutions, have been ramped up ever since, with the strong awareness that pandemic preparedness should be a continuous process and not an overnight reactive response. Soon after emergence of the Wuhan cluster, our local public health services started preparing to tackle this new virus. Countries with recent experience of respiratory pandemics caused by novel coronaviruses (*e.g.,* SARS, Middle east respiratory syndrome [MERS]) had a tendency to have developed public health pandemic response plans and implemented these at an early phase.

COVID-19 is spread mainly through direct contact with airborne respiratory droplets from infected patients, who typically present with a range of respiratory tract symptoms. Asymptomatic carriers contribute to significant disease spread and pose a major hurdle in containing the disease.^3,4^ Chest radiographs are the first-line imaging modality for suspect cases, as well as the follow-up imaging modality to monitor the progression of disease in confirmed cases, although the multinational consensus statement from the Fleischner Society does not recommend daily chest radiographs in stable intubated patients with COVID-19.^5^ Chest CT is reserved for the assessment of subtle radiographical findings, identifying characteristic features, disease prognostication and evaluation of complications such as pulmonary embolism.^6–9^ The American College of Radiology (ACR) strongly discourages performing routine chest CT as a screening tool as a normal chest CT does not mean a person does not have COVID-19 infection and an abnormal CT is not specific for COVID-19 diagnosis. A normal CT should not dissuade a patient from being quarantined or provided other clinically indicated treatment when otherwise medically appropriate.^10^ Some centres have even started on artificial intelligence (AI) initiatives whereby gathered data enable doctors to create simulations, whereby a patient’s CT images could be used to virtually model how patients might respond to treatment.^11^

## The Singapore situation

In Singapore, the epidemic evolved in different patterns. Since confirmation of the first COVID-19 imported case in Singapore on 23 January 2020, there has been a gradual increase in numbers of infected patients, mostly from imports and within small local clusters, in the first 2 months or so, that is February and March 2020. Most of these initial patients were managed at the National Centre for Infectious Diseases (NCID), a 330-bedded purpose-built facility for handling disease outbreaks in Singapore, with each of the other public hospitals receiving relatively small numbers of cases.

**Figure 1.**
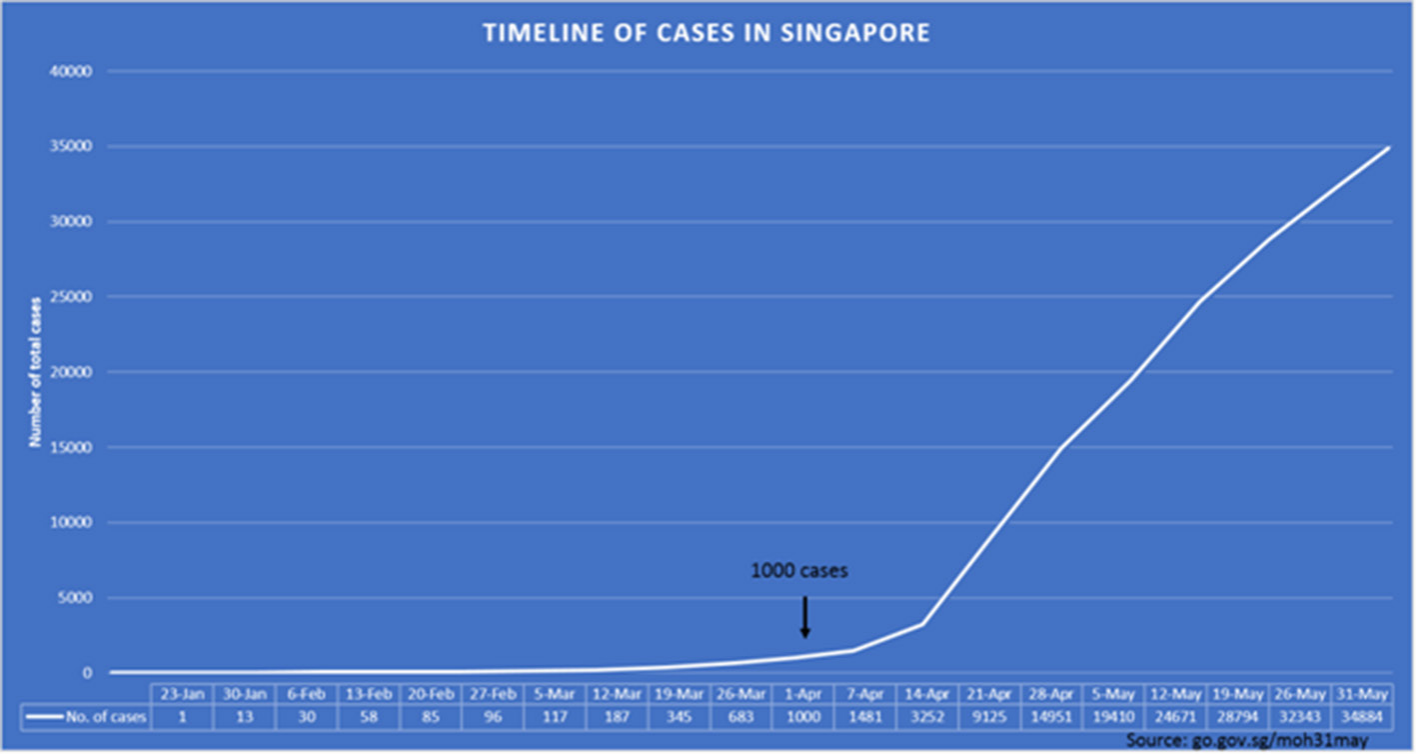
Timeline of COVID-19 cases in Singapore since the first local case on 23 January 2020. The increase in cases from April 2020 onwards is due mainly to detection of large clusters of infections in foreign worker dormitories.

A sudden, unexpected and rapid increase in the number of cases then occurred, rising from a total of 1000 cases on 1 April 2020 to 34,884 cases on 31 May 2020.^12^ Figure 1 shows the timeline of the total number of COVID-19 cases against the salient dates. This sustained spiking over the past 2 months to date were mostly from rapid spreading of infection among dormitory-housed foreign workers. Aggressive testing of residents of these foreign worker dormitories gave rise to the large numbers of positive cases. Although most of those infected were generally young and healthy workers who were asymptomatic or had mild symptoms, this surge in numbers resulted in an overwhelming number of cases seen daily at all public hospitals, including ours, over a relatively short period of time.^13^

## Department situation

While physical separation of facilities and workforce was possible and more readily implementable at larger thousand-bedded tertiary hospitals, it was more challenging in the radiology department of a medium-sized regional hospital such as ours, a 795-bed general and acute care hospital that serves more than 550,000 people living in the northern sector of Singapore, where space within a fixed physical footprint was a constraint. In this article, we share our experience of mitigating and adapting to a rapidly changing pandemic situation and describe how we tried to simplify work processes, and implement and modify operational measures, as the disease unfolded over the past 4 months or so.

## Practical problem-based approach

Our approach was broadly guided by two principles:

1. Patient centric: we focused on managing the increased surge of COVID-19 cases, imaging them safely and protecting our regular patients at the same time. This involved strict infection control measures and adhering to the set practices.
2. Workforce centric: the focus was to preserve our workforce resources, prevent infection transmission to our staff and enforce protective measures.

### Patient centric measures

#### Portable XR machines and CT scanners

**Figure 2.**
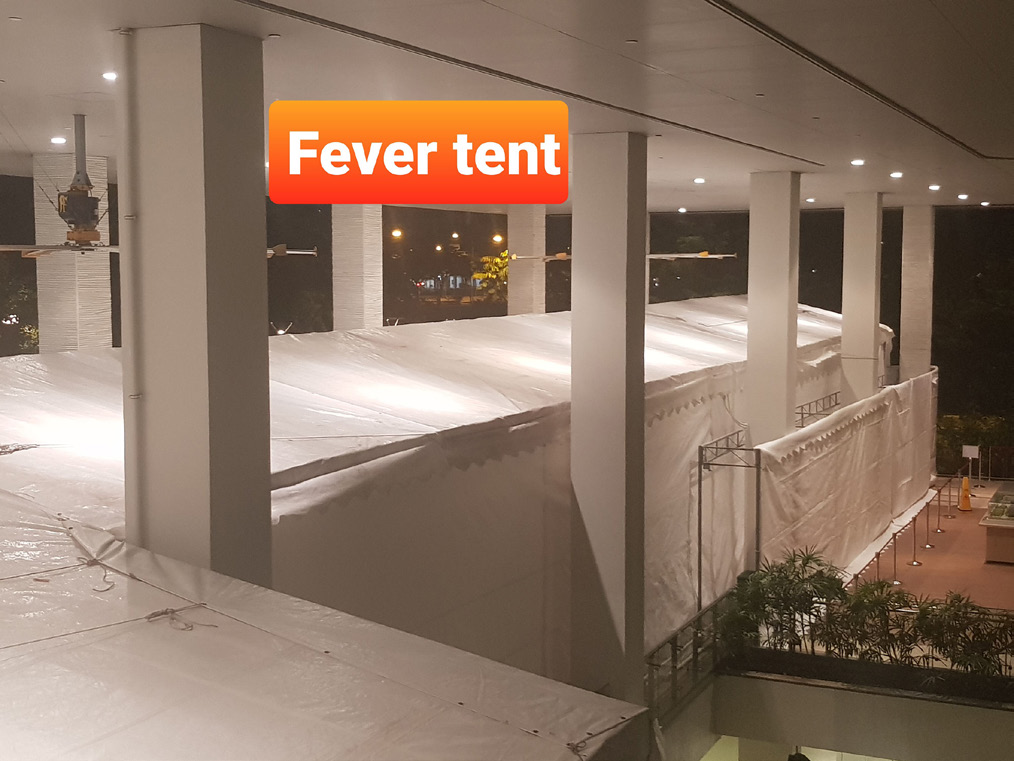
Photograph of the Expanded Screening Wing (ESW)(or “fever tent”) set up in the lobby outside the ED to decant patients more effectively and reduce congestion within the ED

From the outbreak onset, measures were taken to keep all potential COVID-19 cases presenting to the emergency department (ED) separate from the “cold” patients and HCWs, in order to prevent cross-infection. Initially, these COVID-19 suspect cases were seen in the isolation wing of ED which had only nine rooms. To minimize potential cross-infection, portable radiographs were done for this group of patients if chest radiographs were required. Due to anticipated space limitations, planning for separate “fever tent” started on 24 January 2020 and the tent, known as the Expanded Screening Wing (ESW), became operational on 7 February 2020. The ESW was located adjacent to the ED (Figure 2).

**Figure 3.**
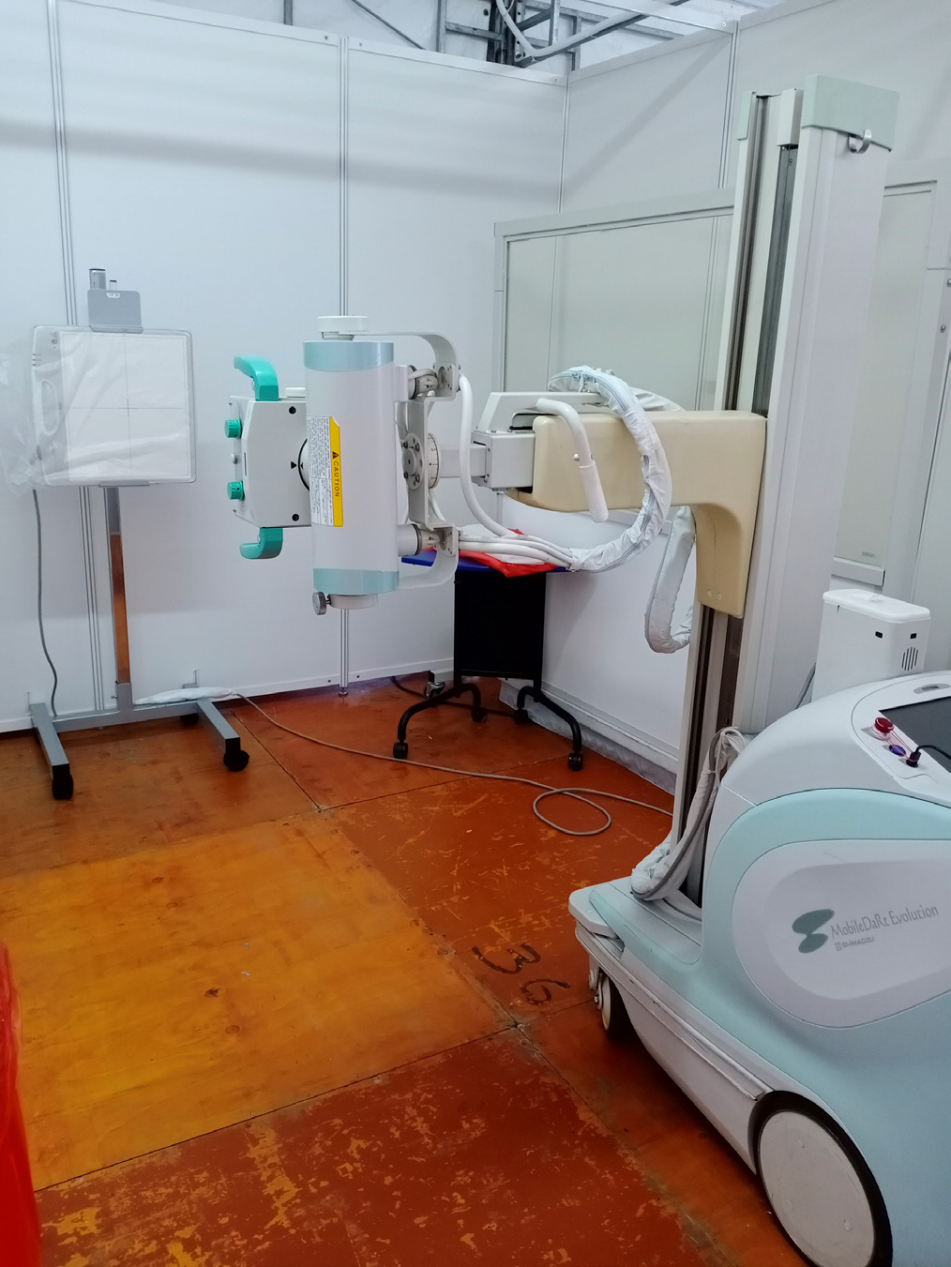
Photograph of the temporary X-ray cubicle within the ESW shows a portable DR X-ray machine and adjacent lead shield. The X-ray detector is covered with disposable plastic and cleaned after every patient

Initially, we deployed a portable X-ray machine that used a computed radiography (CR) cassette. However, when the ESW workload started to increase, we switched to a portable digital radiography (DR) X-ray machine. The use of this DR unit helped speed up the turnaround time (TAT) for chest radiographs in suspected COVID-19 patients. The radiographs were performed in a make-shift X-ray cubicle with a lead shield placed on one side of the cubicle. The X-ray detector was covered with a disposable plastic sheet and the machine was wiped down with 70% isopropyl alcohol (Figure 3). The agreed-upon TAT for chest radiograph was a maximum of 60 min from the time of radiography request to completion of the radiological report. Priority for reporting was given to this group of patients to assist in the ED physician’s decision-making whether to up-triage or deisolate each patient. A senior radiologist was always also available (24 h daily, including weekends) to render a second opinion, upon request by clinical colleagues. This was particularly useful when screening many suspected patients, as positive findings on chest radiographs allowed quick isolation and contact tracing for the patient, even before the polymerase chain reaction (PCR) test results became available.

In our hospital, we have a short stay ward to accommodate ED patients whose lives were not in immediate danger (P2 patients). These patients stayed for a maximum of 23 h, while they were investigated and/or observed, after which they were either discharged or admitted. To accommodate P2 patients who came in with respiratory symptoms suspicious of pneumonia, the hospital set up an acute respiratory wing (ARW) within the current short stay ward, which became operational on 11 February 2020. Patients on the way to the ARW stopped over at the ED P2 radiography room to get their radiographs done, as a time-saving measure. With the increasing number of patients being sent to the ARW, on 27 March 2020, a portable X-ray machine was parked in the corner of the ARW itself, to facilitate chest radiographs and other simple radiographs for these patients. This aimed at preventing unnecessary movement of patients into the clean zone in the ED P2 area. One radiographer was deployed at the ARW full time.

Once the nasal swab PCR results were available and positive for COVID-19 and/or initial chest radiograph done in the ED showed features of pneumonia, these patients were isolated and admitted. All subsequent follow-up radiographs for these patients were performed by regular inpatient radiographer team in the isolation wards or intensive care unit (ICU) as portable radiographs.

We have three CT scanners, one located in the ED and two located in the main department that is sited on a different level in the hospital. Our hospital does not have portable CT scanners and it was not considered a readily feasible solution, due to financial and logistical constraints. Due to current existing workload, we were not able to free up a CT scanner solely for COVID-19 patients, despite a marked reduction of number of the outpatients, particularly for clinical conditions which can be deferred. We, however, modified our pandemic response protocol and identified one of the inpatient CT scanners (“hot scanner”) for imaging warded COVID-19 confirmed cases. CT was performed only in selected cases after discussion between the radiologist and the physician-in-charge.

**Figure 4.**
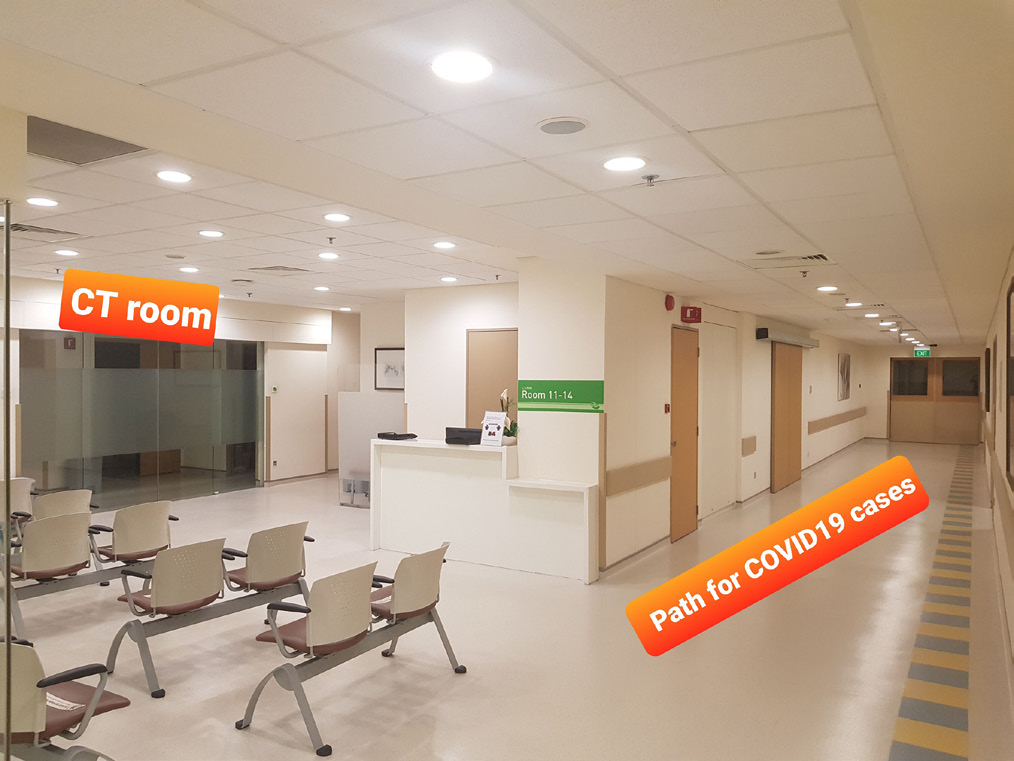
Photograph shows the separate entrance and path for transport of COVID-19 confirmed cases to the CT scan room in main radiology department. The waiting areas used by regular outpatients during office hours are cleared before a positive case is brought in

We aimed to use the CT scanner in the main Department of Diagnostic Radiology (DDR), nearest to the isolation wards and ICU housing the COVID-19 patients as the “hot scanner.” We also made sure that the route used to bring these patients to the radiology department was separate from the route used by non-COVID-19 patients (Figure 4), in order to minimize cross-infection with regular outpatients and inpatients coming to the department. To optimize use of all our scanners and maintain the TAT, we had dedicated time slots to perform COVID-19 confirmed cases as follows:

1. COVID-19 confirmed inpatient/outpatient cases to be performed in one of the main DDR scanners during a dedicated 4 h time slot at the end of the day.
2. COVID-19 confirmed cases in ED coming from the ESW or ARW to be scanned in ED CT scanner if the ED is not performing any urgent ED scan or backlog is not high. However, if ED scanner is busy and the COVID-19 positive scan is deemed urgent, it can be performed in main DDR, if slot available.

A caveat is that any emergency case takes priority and is scanned as soon as possible in whichever CT scanner is available followed by terminal cleaning of the room. This was achieved by using diluted bleach solution (6 mg chlorine-releasing disinfectant tablet added to 1000 ml of water) to wipe down the machine, floor and walls of the scan room.

For COVID-19 positive patients who needed imaging for other clinical conditions, similar principles of infection control were applied for subspeciality scanning such as ultrasound, CT, nuclear medicine and magnetic resonance imaging (MRI), as well as for interventional procedures. For ultrasound and MRI, we reserved dedicated slots at the end of each afternoon for COVID-19 positive cases following which the machines and room underwent terminal cleaning. Sonographers performing the scan follow standard infection control protocol and don full personal protective equipment (PPE).

Within the department, we ensured that safe distancing measures were put in place, such as minimum 1 m distancing between chairs in the waiting areas. Only one family member or caregiver was allowed to accompany each patient with special needs entering the department. All patients and visitors entering the hospital premises and the radiology department had to undergo mandatory temperature screening and fill up a screening form providing standard information mandated by the MOH. All group meetings were cancelled, for example public seminars, patient focus groups and patient family conferences.

#### Infection control

A two-pronged approach was employed for infection control and staff protection. Staff self-hygiene had always been continuously emphasized as part of our hospital culture. All staff underwent regular audits of hand hygiene, with compliance data for each department published as one of the items for the hospital’s quality indicators. This has ensured a heightened sense of the importance of personal hygiene, particularly hand washing, among all staff.

Secondly, the availability of PPE, such as fluid-resistant surgical masks, N95 respirators and surgical gowns was crucial. Since the SARS outbreak, the national stockpile of PPE is sufficient to sustain frontline staff for 6 months, in line with the pandemic response plan published in 2004 by our MOH.^14^ There were regular mask-fitting exercises held for all staff, so that updated staff records of N95 mask sizes and types could be maintained. All staff were fitted for at least two different brands of N95 masks so that if supply of one brand is limited, our HCWs were not left without protection. From the start of the COVID-19 outbreak, both fluid resistant surgical and N95 masks were distributed to staff daily at designated sites to ensure accountability and prevent misuse or wastage of resources. Staff were required to complete e-learning modules on handling of droplet infections, suspected patients and PPE donning/doffing procedures. Regular update e-mails from the hospital infection control unit and the hospital intranet were other sources of vital information. It was mandatory for all staff to wear fluid-resistant surgical masks when performing their duties in clinical areas. The use of N95 respirators, disposable gloves with over gown cuffs, eye protection (such as face shields or goggles), hair nets and shoe covers was required when caring for suspected or confirmed COVID-19 patients.^15^

Mandatory bd temperature screening and recording were implemented among HCWs, regardless of whether they were on or off duty. As part of MOH guidelines, everyone (including HCWs) with acute respiratory infection symptoms presenting to general practitioners or family physicians were given a mandatory 5 days of medical leave and instructed to stay home. For unwell staff with higher risk to possible COVID-19 exposure, they were to report to our hospital ED so that swab testing can be performed. These measures aimed to detect disease early so that the affected staff can be isolated and managed separately, without infecting other HCWs and patients.

### Workforce centric

#### Workforce demands and mitigating steps

There was added stress on existing radiology workforce with the increased demand for imaging during the COVID-19 pandemic. Multiple factors contributed to the situation: (a) additional workload of screening chest radiographs in the ED (ESW and ARW), as well as the isolation wards; (b) increased time required to perform cross-sectional imaging on COVID-19 positive or suspected cases due to additional infection control measures; (c) less available workforce due to segregation strategies to protect staff providing essential services and redeployment of staff to other areas of need; both within our hospital and at other institutions, for example, NCID and (d) limited hospital beds.

As the disease spread and number of suspected COVID-19 patients increased, our hospital had to rapidly respond and adapt to the situation. There was an urgent need to diagnose and discharge non-COVID-19 patients to free-up bed capacity. The hospital took certain decisive steps to mitigate the issue: (a) almost 70% of the existing hospital beds and the adjoining community hospital were converted to COVID-19 ready wards; (b) the ED P3 area patients (stable patients who were deemed “cold” after triaging) were moved to the specialist outpatient clinic (SOC) block, labelled as ambulatory emergency clinic (AE clinic) to increase holding capacity for acute and ”‘hot” cases in the main ED; (c) outpatient attendance at our SOC was drastically reduced by non-acceptance of external referrals (except for those deemed urgent) and cancelling/postponing appointments and use of teleconsultations for existing outpatients; (d) cancellation and postponement of elective surgical and interventional procedures, and (e) converting operating theatres to temporary ICU.

From end January 2020, all HCWs were heavily encouraged to cancel or defer non-essential overseas travel by our hospital. As the number of cases increased across the region by mid-February 2020, MOH initially froze all leave for HCWs till the end of March 2020, which was extended further till the end of the year as of mid-March 2020. Cancellation of leave was intended to ensure the availability of HCWs. This also reduced the potential risk of exposure to the disease during overseas travel. The staff were given the option to either carry forward their leave to next financial year or get a one-off encashment at the end of the year. Local leave was however allowed, so that staff could be recalled to work if required by service needs. The hospital considered reimbursement of costs incurred as a result of travel cancellation on a case-by-case basis. Staff who were currently on overseas fellowship training, study trips or group training were recalled before international travel embargoes took effect.

#### Focused imaging protocols

Working with our clinical colleagues, postponement and cancellation of non-urgent outpatient imaging helped reduce the workload for radiology staff. This was also in line with the national approach to restrict non-essential local travel for the public. Essential imaging and procedures continued, including urgent or emergency procedures performed for ED patients as well as inpatients; and urgent outpatient procedures, such as those performed for cancer diagnosis, treatment and follow-up. We anticipated a steep increase of these non-urgent procedures once the pandemic situation is controlled, resulting in longer wait time for imaging/procedures. Hence, during the earlier phases of the outbreak, we managed to expedite completion of most of these outpatient scans scheduled within a 2-week period. Additional staff (radiologists, radiographers, nurses and other radiology department support staff) were rostered over the weekends to clear the extra workload.

#### Staff safety

We introduced some workforce practices to enhance safety for our staff. In the ESW, a single radiographer, with full PPE and a portable X-ray machine, performed all the radiographs for a cohort of patients and processed the radiographs. In this way, we did not risk exposing multiple radiographers over a certain time period and at the same time also saved on PPE usage.

For cross-sectional imaging, the scans were performed by two radiographers. The radiographer who had direct contact with the patient was deemed to have a higher potential risk of contamination and/or exposure to the virus. This radiographer donned the full PPE, adjusted patient positioning before scan, and moved the patient out of the scan room after the scan completion. Subsequently, this radiographer also cleaned the machine and related equipment. The second radiographer, who was inside the control room, was not directly exposed and the control room remained clean. The two radiographers swapped roles for alternate patients.

For suspected or COVID-positive cases, single-use disposable plastic sheets were used to cover the portable X-ray machine or CT scanner. After imaging was performed, the machines were wiped down with 70% isopropyl alcohol wipes. Terminal cleaning of the CT scan room was usually done by our housekeeping staff, using diluted bleach solution (6 mg chlorine-releasing disinfectant tablet to 1000 ml of water) to wipe down the walls and floor. After this, the scan room was left to dry for 45–60 min before the next patient could be scanned. Terminal cleaning had to be performed as we do not have a negative pressure CT scan room. The protocol for machine disinfection was according to recommendations by the respective vendors, and further approved by the hospital infection prevention committee.

#### Staff cohorting

Cohorting strategies for all radiology department staff were put in place to reduce the risk of human-to-human transmission among staff and to preserve the department’s capability to meet the hospital’s radiology needs, should one team/group be required to be quarantined. Cohorting could be temporal (by time) or spatial (by physical location). The differences between the two is beyond the scope of this article and has been described by others.^16^

During the initial stages of the outbreak, almost all suspect cases underwent a chest radiograph with very few cases requiring CT to exclude other pathologies like pulmonary embolism. However, as the pandemic worsened and the number of confirmed cases increased markedly as shown in Figure 1, large numbers of patients were housed in temporary community care isolation facilities. As of 31 May 2020, there were 13,616 active cases, of which 13,242 cases had mild symptoms or were clinically well and were being isolated at these community care facilities.^13^ We started to get more requests for CT from the ED for COVID-19 unrelated conditions for this group of patients, which are otherwise deemed routine requests from ED during non-pandemic period such as CT abdomen and pelvis scan to rule out acute cholecystitis or acute appendicitis, CT/ MR brain for stroke or US testis to exclude torsion. As we adapted to this sudden change in ED requests for speciality modality imaging, we realized that there was a concomitant increase in the request for non-urgent inpatient scans as well. This was mainly to expedite discharge of inpatients who are relatively stable and create bed space for COVID-19 cases who were moderately or severely ill.

**Figure 5.**
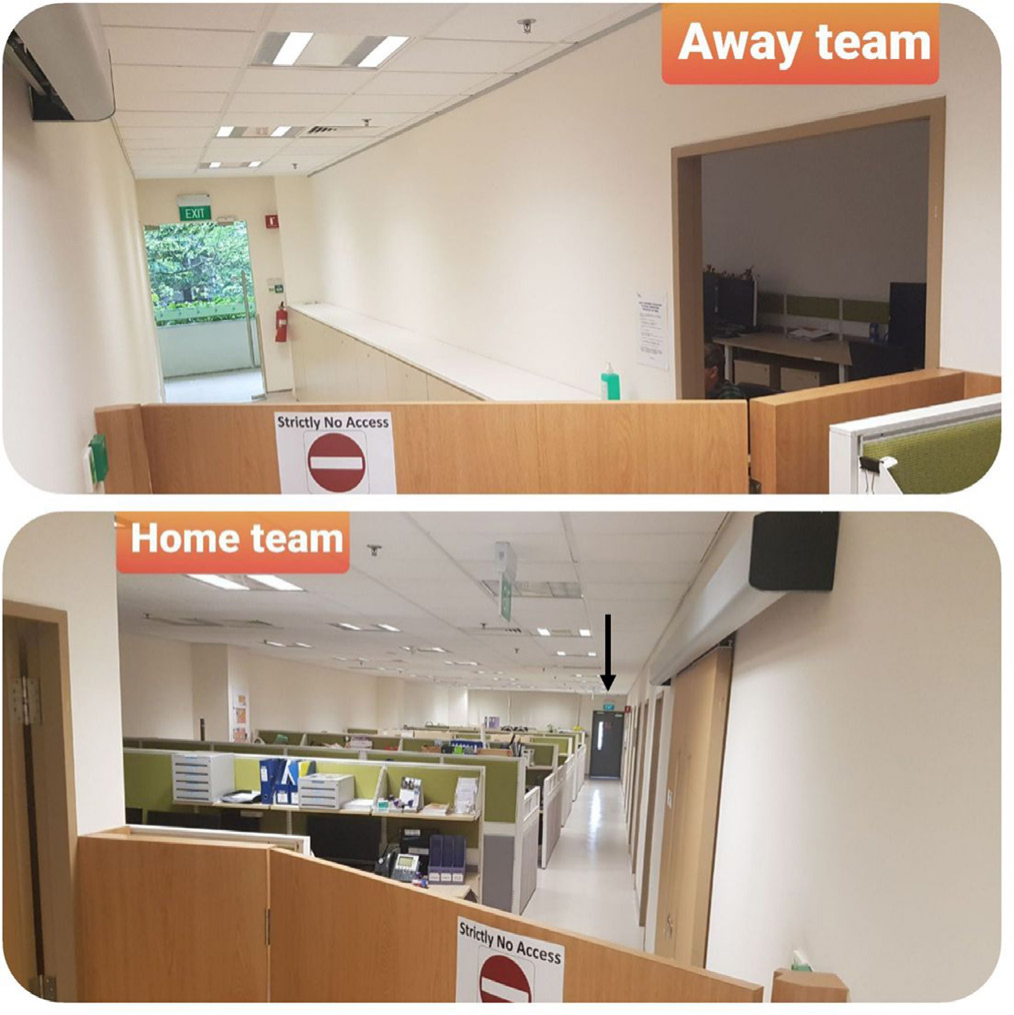
Photograph shows the newly constructed 1.6m high wooden wall to cohort radiologists into “home” and “away” teams. This wall separated the central reporting room from the rest of the main department. The “away team” gained entry via the glass staff entry door at the end of the corridor. The internal door built within the wall was permanently closed but could be opened (shown in second image) in an emergency to enable rapid and urgent movement of staff in or out of the department, in case of any patient emergencies *e.g.* code blue collapse or general emergencies *e.g.* fire alarms. The second image also shows the other staff entry door (black arrow) at the other end of the corridor for the “home team”

In anticipation of disease progression, our department implemented staff cohorting on 2 March 2020. At that time, there was still a heavy outpatient and elective inpatient workload. We opted for a hybrid system with the department, due to staffing constraints. The diagnostic radiologists were spatially cohorted into two equal teams, each led by a senior radiologist. Both teams had equal division of workforce with regard to subspecialty skillsets and distribution of seniors and juniors. Due to physical space limitations, one “away team” were located outside the main radiology department, occupying the satellite radiology sections at the ED, specialist outpatient clinic (SOC) block, adjacent community hospital and a large central reporting room that opened onto a staff corridor leading to a staff entry door to the main radiology department. A 1.6 m high wooden wall with an internal door was installed within the department to separate the central reporting room from the rest of the main department(Figure 5). The internal door was permanently closed but could be opened in an emergency to enable rapid and urgent movement of staff in or out of the department, in case of any patient emergencies, for example, code blue collapse or general emergencies, for example, fire alarms. The “home team” were stationed in the rest of the main department, with access either through the main department entrance or the other staff entry door at the other end of the staff corridor. Some reporting workstations had to be relocated to ensure an equal distribution between the two teams of diagnostic radiologists.

The rest of our department was cohorted temporally. The diagnostic radiographers at ED were divided into four teams, each team working two consecutive 12 h shifts (800am–800pm or 800pm–800am), followed by two rest days each. The radiographers in the CT and MRI scanning rooms were split into two teams, each working for two days (800am–600pm, with extension to 800pm daily if needed), followed by two rest days. The two interventional radiology teams (each consisting of interventional radiologists, radiographers, nurses and healthcare assistants) covered all interventional radiology work for 24 h (800am–800am) for two consecutive days, followed by two rest days. The remaining radiographers (ultrasound, nuclear medicine) and administrative staff were deemed at low risk of exposure and were not segregated.

With markedly worsening of the COVID-19 outbreak at the beginning of April, the Singapore government implemented a partial lockdown, called “circuit breaker” measures, initially from 7 April 2020 for a month till 3 May 2020 and recently further extended to 1 June 2020.^17^ With marked decrease in both elective outpatient and inpatient imaging, in particular musculoskeletal MRIs, and increase in available radiologists due to cancellation of leave, and return of our radiologists who have served their post-overseas travel 14 day stay home notices, our diagnostic radiologists were able to move to a partial temporal segregation timetable starting from 4 May 2020. Our diagnostic radiologists were split into three teams: one small permanent daytime team and two shift teams. The small permanent daytime team consisted of three senior radiologists covering three main subspecialty areas of neuroradiology, body imaging and musculoskeletal radiology; and four breast radiologists and nuclear medicine specialists, as breast and nuclear medicine imaging were all done during regular office hours. The daytime team (working 800–530pm daily) were separated by physical location from the two shift teams. The two shift teams were separated temporally, and each worked 12 h shifts (800am–800pm daily) for 2 days, followed by 2 days of rest. Each shift team had equal division of workforce with regard to subspecialty skillsets and distribution of seniors and juniors. To cover overnight work (800pm–800am) daily, there was a small on-call team comprising three radiologists from all teams, with all individuals separated physically when on duty.

The option of working from home (WFH), in short teleradiology, was not mandated by hospital management in public healthcare institutions. This was following termination of direct access to the internet from hospital’s internal systems by the Singapore government following previous cyber-attacks.^18^ However, it was still possible for our radiologists to access images using their personal computers via virtual private network (VPN).

Following extensive discussion among our senior radiologists, most preferred to come to the radiology department to work and report. There are however inherent advantages of WFH, for example, personal protection of individual staff and increased segregation. We have kept the option to report from home open for those who wanted to try it out, although there are currently no volunteers. There were however multiple considerations and limitations to take note of. The Table 1 lists the requirements and limitations of WFH and ways of mitigating them.

**Table 1.** Limitations and mitigating steps for working from home

Limiting factor	Requirements to mitigate
System requirements	Updated hospital laptop Working VPN access Compatible RIS/PACS software installed. Mini display port cable
Internet requirements	Broadband internet Fast WiFi or direct LAN cable connection
Hardware (monitor)	Size: 27 inch (minimum) Resolution: QHD (2560 × 1440), 4K (3840 × 2160) recommended Type: In plane switching (IPS) recommended. No twisted pneumatic (TN) Connection: Display port for 4K, HDMI/display port for QHD Contrast at least 1:1000 Brightness: 350 cd/m^2^
Limitations	No speech mike (institution specific) eRad cockpit on different interface (institution specific) Inability to report radiographs and mammograms Some cross-sectional studies like CT colonography, CT coronary angiograms, MR cardiac, dual energy scans cannot be reported off-site (additional post-processing software required). Checking of certain ultrasound exams by radiologists is mandatory, for example, specialized musculoskeletal studies. Limited IT support at home.
Workflow	Must attend phone consults, review images on PACs when required Confidence and responsibility of reports from radiologists (possibility of errors due to technical factors must be borne on mind) Normal workflow distribution with other team members, for example complex ultrasound, mammograms. May need to return to department when required, for example technical issues like VPN downtime or reviewing images on machine-specific post-processing software.

HDMI, high-definition multimedia interface; LAN, local area network; MR, magnetic resonance; QHD, quad high definition; RISPACS, Radiology Information System/Picture Archiving and Communication System;VPN, virtual private network.

#### Other considerations of segregation

The interhospital visiting consultant scheme had been suspended since 27 February 2020.^19^ Transfer of residents in training between institutions have also been suspended. To cater for their training needs, we leveraged on teleconferencing technology for intradepartment learning sessions as well as webinars. We also tried to provide as much teaching and training as possible within the segregated teams, with rostering being tailored to their subspecialty areas of residency rotation.

Interdepartment interactions between radiologists, radiographers and other staff of the department were minimized, and restricted to non-physical forms of communication as much as possible, for example, sharing of messages and scanned request forms through electronic notes on RIS and annotated images on PACS. Each team leader disseminated the information to the rest of the team via group messages or emails. Regular face-to-face department meetings were suspended and if a physical meeting was necessary, standard social distancing policy was strictly complied with. We have made use of the Zoom application for department meetings and for intrainstitutional and interinstitutional meetings, as well as conducting job interviews.

#### Psychological wellbeing of staff

In these challenging and unpredictable times, our staff were faced with tremendous physical and mental burden. Many of our international staff have not had physical contact with their families residing overseas for months. To boost staff morale, the department and the hospital have taken up many initiatives, such as providing lunch and dinner to all frontline staff, including our radiographers and nurses, working over the weekends and on public holidays. The hospital has tried to help staff in various ways, for example arranging childcare for staff with small children that have lost their normal caregivers because of movement restrictions between households, refunds for cancelled overseas trips, arranging for alternative local living quarters for our Malaysian staff who previously commuted daily from our neighbouring country. The psychological medicine department has a psychological support program called peers around lending support (PALS), available to all staff to address any psychological issues and provide counselling services. Staff members were regularly reminded of the availability of PALS.

## Lessons learnt and direction for the future

The current pandemic had taught us some important lessons that made us rethink the design layout of the radiology department. Disaster planning and business continuity contingency plans should be incorporated in department design and layout.

In a new department, the workforce is expected to be modest. For a lean team, temporal cohorting is extremely difficult to implement without compromise on radiology service coverage. Keeping in mind the option of spatial cohorting, the department needs to be planned such that in the event of any future outbreak or pandemic, it is easy to divide the reporting areas into teams. Satellite reporting rooms in outpatient, inpatient as well as ED, administrative areas, conference rooms and meeting rooms, should have provisions for installing additional reporting workstations.

During spatial cohorting, reporting stations that are near high patient flow, high acuity and emergency areas should be designated as “high risk” areas. The other workstations can be designated as “low risk.” At-risk radiologists (pregnant or older than 60 years old) should work in the “low risk” areas as far as possible.

The ability to report remotely is useful not just during pandemics but also in non-pandemic periods, when subspecialty opinions can be sought after office hours. However, there are some intrinsic issues pertaining to remote reporting. Firstly, cybersecurity is a growing problem in healthcare and past security breaches, both locally and globally, have led to leakage of sensitive patient data. Even if we can get around the cybersecurity issues, there are still cost, maintenance and support issues that come with installing workstations at home. Leasing of these equipment may be an option to consider.

Negative pressure procedure rooms should be planned for imaging modalities such as CT and MRI, as well as the angiography suites. A dedicated ultrasound unit should be set aside to perform portable bedside ultrasound for isolated patients.

Timely and accurate information dissemination is critical during outbreaks and times of crisis. Misinformation leads to low staff morale and can also put our healthcare professionals at risk. A monitor can be put up in key areas like the ED reporting room and inpatient CT room, which can serve as a live dashboard notifying staff about confirmed or suspected cases been scheduled for procedures. Similar dashboard features have already been in place within ED.

Innovative ways of performing portable radiographs should be considered and implemented if deemed safe. One option is to utilize a robotic-assisted mobile X-ray unit for intensive care and isolation cases. The nurses in the ward, who are already donning PPE can help in positioning of the patient while the radiographer remains outside the room. This avoids direct contact between the radiographer and the patient, as well as reduces usage of PPE. In the USA, Penn State Health Medical Center has installed drive through portable X-ray units at the entry gate.^20^

Frontloading of the screening chest radiograph has multiple advantages. Firstly, it helps in further triaging patients based on the radiographical findings, for example, patients with positive radiographical findings get isolated and swabbed earlier than the asymptomatic cases. Secondly, it saves workforce and other resources such as PPE, as all one needs is a sole radiographer manning the unit donned in PPE. There is less time and resources spent on decontaminating a portable unit as there is less patient contact and time is saved in doing air exchange and wipe down. Thirdly, regular mobilization of the unit causes significant low-grade blunt injury to the radiographers like backache which can be avoided with a drive through unit. Lastly, during non-pandemic times, this unit can be used to frontload many of the radiographs done in ED.

## Conclusion

During a pandemic, key factors are sustainability of mitigating measures and the ability of a radiology department to adapt quickly. The department must protect its staff and the vulnerable population while promptly providing essential services. What may seem like the correct strategy today may be superseded by another course of action in an ever-changing scenario. We described how our department shifted from an initial spatial cohorting plan to a temporal cohorting plan with hybrid elements, as the outbreak progressed. This was achieved without compromise to patient and staff safety. The aforementioned strategies and workflows are gleaned from our experience as a radiology department in a medium-sized regional hospital with space constraints. We acknowledge that some of these measures may not be applicable or appropriate in other departments due to inherent differences. However, we believe that the underlying basic principles are uniform and if implemented, or adopted in a modified manner, can still be relevant to many radiology departments during the current COVID-19 pandemic, particularly in small-sized and medium-sized hospitals.
